# Isolated Non-displaced Pisiform Fracture: A Diagnostic Challenge in Ulnar-Sided Wrist Trauma Managed Conservatively

**DOI:** 10.7759/cureus.87637

**Published:** 2025-07-09

**Authors:** Amir F Amer, Turki M Alrehaili, Muaath M Alrehailiy

**Affiliations:** 1 Orthopedic Surgery, International Medical Center, Jeddah, SAU; 2 Orthopedics, King Fahad Military Medical Complex, Jeddah, SAU; 3 Orthopedic Surgery, Al-Noor Specialist Hospital, Makkah, SAU

**Keywords:** carpal injuries, conservative management, pisiform fracture, ulnar-sided wrist pain, wrist trauma

## Abstract

Carpal bone fractures are uncommon fractures, with pisiform fractures being exceptionally rare. This case report describes a 29-year-old male military member who sustained a non-displaced pisiform fracture after falling approximately six steps. The patient presented to the emergency department with localized left wrist pain and mild swelling over the pisiform bone, without other associated injuries. Initial radiographs, including anteroposterior, lateral, and oblique views, confirmed a transverse non-displaced fracture of the pisiform. The patient was managed conservatively with a below-elbow ulnar gutter splint in mild palmar flexion for two weeks, followed by progressive mobilization. At follow-up, radiographs demonstrated early signs of healing, and the patient regained full wrist range of motion without complications by four to six weeks post-injury. This case underscores several key points: (1) the importance of clinical suspicion for pisiform fractures in patients with ulnar-sided wrist pain following trauma, (2) the utility of standard radiographs in diagnosis when multiple views are obtained, and (3) the effectiveness of conservative management with short-term immobilization for non-displaced fractures. Given the rarity of isolated pisiform fractures and their potential for missed diagnosis, clinicians should maintain a high index of suspicion to ensure timely treatment and prevent long-term complications such as chronic pain or osteoarthritis. The successful outcome, in this case, supports current literature advocating for conservative approaches in uncomplicated pisiform fractures.

## Introduction

Carpal bone fractures account for approximately 6% of all fractures, with scaphoid fractures being the most prevalent (70-80%), followed by triquetral (7-8%) and trapezium (5%) fractures [1]. Among these, pisiform fractures are exceptionally rare, representing less than 0.2% of all carpal fractures and often occurring in conjunction with other injuries, such as distal radial or carpal fractures [2,3]. The pisiform bone, a sesamoid bone embedded within the flexor carpi ulnaris (FCU) tendon, plays a critical role in wrist biomechanics. It acts as a fulcrum for force transmission from the forearm to the hand and stabilizes the ulnar column by preventing triquetral displacement [4]. Despite its small size, the pisiform’s functional significance underscores the importance of timely diagnosis and management of its fractures.

The rarity of isolated pisiform fractures poses diagnostic challenges. These fractures are frequently overlooked due to their subtle clinical presentation and the overlapping of adjacent carpal bones on standard radiographs [5,6]. Misdiagnosis can occur when the fracture line is obscured by the triquetrum or other structures or when more conspicuous injuries divert attention [7]. Advanced imaging modalities, such as computed tomography (CT) or magnetic resonance imaging (MRI), are often necessary to confirm the diagnosis, particularly when initial radiographs are inconclusive [8]. The clinical suspicion for pisiform fractures should be high in patients presenting with ulnar-sided wrist pain following direct trauma or hyperextension injuries, as these are common mechanisms of injury [9].

The pisiform’s unique anatomical position and its integration into the FCU tendon make it susceptible to specific injury patterns. Two primary mechanisms have been proposed: (1) direct trauma to the hypothenar eminence with the wrist in hyperextension and forearm pronation, and (2) avulsion fractures caused by the FCU tendon during a fall on an outstretched hand (FOOSH) [5,10]. Repetitive stress can also lead to microfractures, which may progress to complete fractures if untreated [11]. Given these complexities, a thorough clinical examination combined with targeted imaging is essential for accurate diagnosis.

Delayed or missed diagnosis of pisiform fractures can result in complications such as malunion, non-union, chronic pain, and pisotriquetral chondromalacia [3,5]. These sequelae may impair wrist function, weaken grip strength, and necessitate surgical intervention, such as pisiform excision, which carries risks of ulnar nerve neuropathy and reduced wrist mobility [12]. Therefore, early recognition and conservative management, typically involving immobilization in a below-elbow cast for four to six weeks, are critical to achieving optimal outcomes [2,13].

This case report highlights the diagnostic and therapeutic challenges associated with isolated pisiform fractures. By presenting this patient with a non-displaced pisiform fracture and the management approach used, we aim to raise awareness of this rare injury and emphasize the importance of a high index of suspicion in clinical practice and a correct managerial approach.

## Case presentation

A 29-year-old Saudi male soldier fell from a height of six steps and presented to the ER at the National Guard Hospital in Al-Madinah. The patient did not report any specific mechanism of injury, but did report pain over the ulnar side of the left hand with mild swelling. Physical exam showed point tenderness over the volar and dorsal aspect of the pisiform bone without any other swelling or discomfort in the body.

The patient was fully conscious and had appropriate orientation. Assessment of both active and passive range of motion of the wrist showed full flexion and extension without any pain. Likewise, finger flexion, extension, and opposition were present and pain-free. Mild tenderness and swelling over the pisiform region were palpated, but no crepitus was detected.

**Figure 1:**
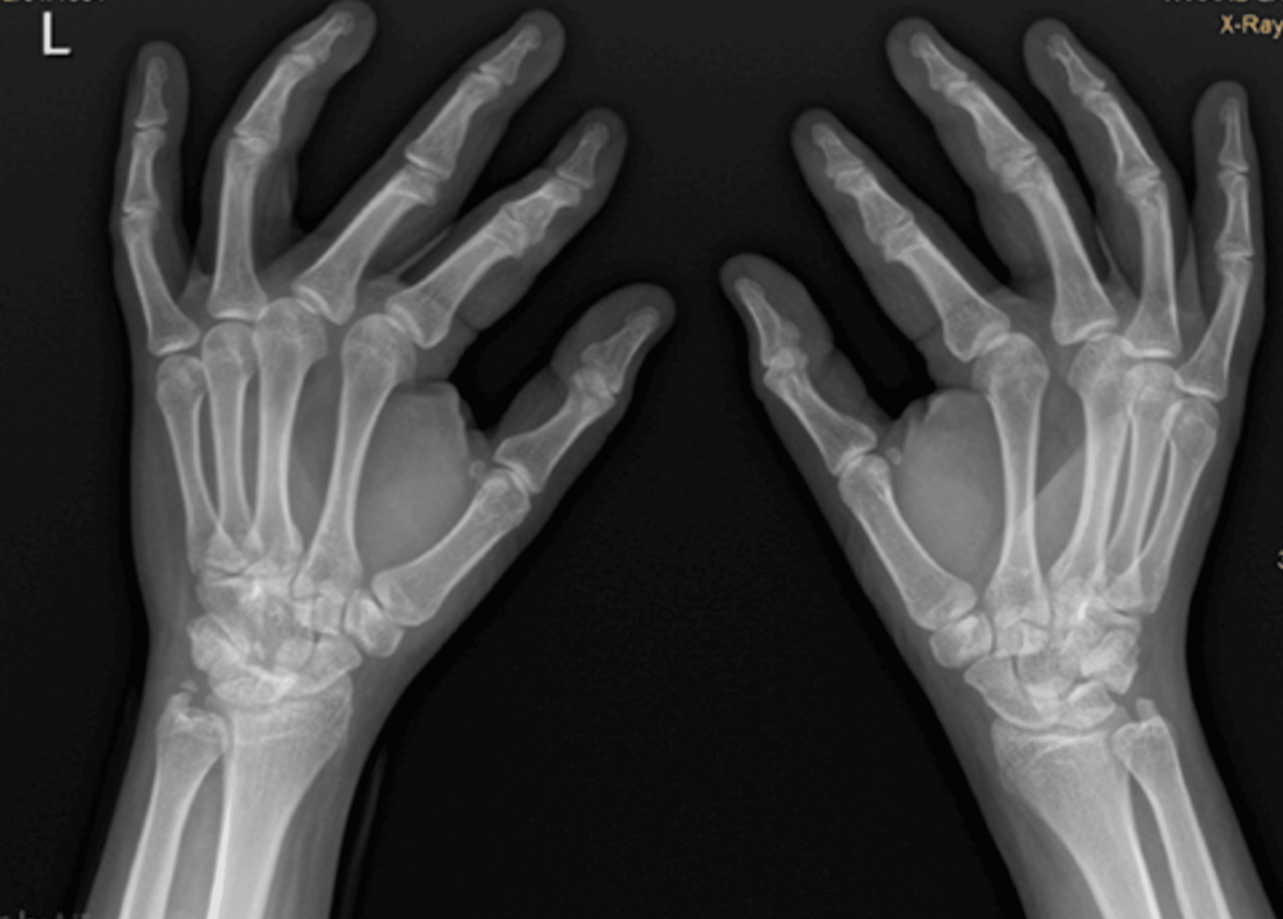
X-ray of the left hand in lateral view.

**Figure 2:**
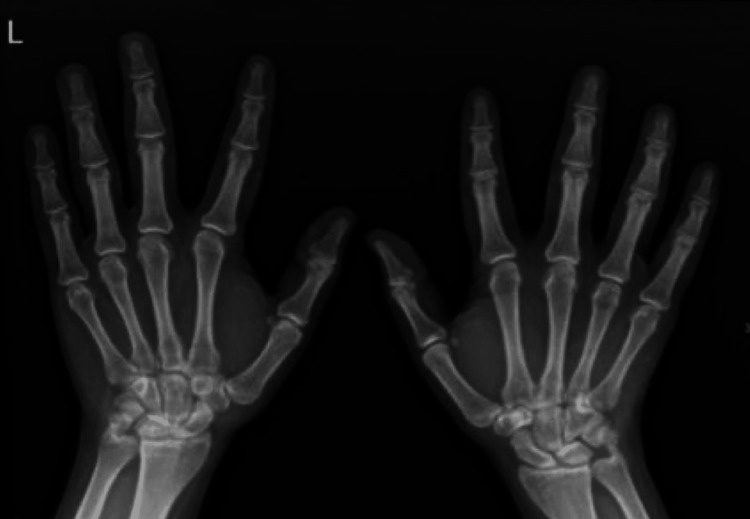
Posteroanterior X-ray view of the left hand.

Initial radiographic evaluation included standard anteroposterior, lateral, and oblique views of the left wrist. The X-rays revealed a non-displaced transverse fracture of the pisiform bone (Figures 1-2). No associated fractures or ligamentous injuries were identified. Due to the subtle nature of pisiform fractures, additional imaging such as a CT scan was considered, but in this case, it was unnecessary because the fracture line was clear on plain film radiographs.

Conservatively, the patient was treated with a below-elbow ulnar gutter splint set in slight palmar flexion to further reduce strain on the pisiform. Careful attention was made during the application of the splint on the metacarpophalangeal (MCP) joints so that the fingers would remain free for movement. Instructions were given for follow-up as an outpatient after discharge.

**Figure 3:**
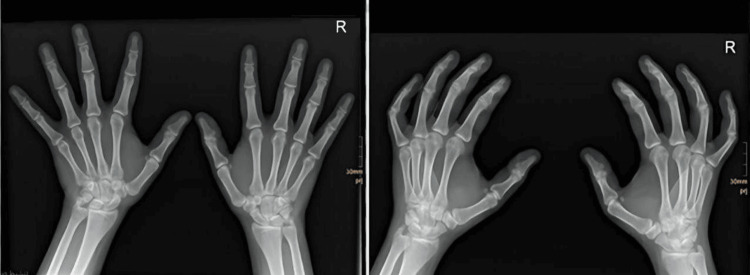
X-ray of the left hand showing early signs of healing.

During the two-week follow-up appointment, the patient’s splint was removed, and repeat X-rays taken showed signs of fracture union (Figure 3). The patient was clinically observed to have considerable pain relief and only slight tenderness around the pisiform. Swelling was significantly less, and wrist and finger range of motion was full and pain-free.

Physiotherapy aimed at restoring strength and flexibility was initiated as part of the rehabilitation. The patient returned to pre-injury activity levels and was functionally fully active by the four- to six-week follow-up with no sustained injuries or chronic pain. There were no noted complications of malunion or sustained pain.

## Discussion

The clinical presentation in this case - a 29-year-old male military personnel with a non-displaced pisiform fracture - is indeed a clinical rarity. As it is noted, the anatomical position of the pisiform bone, as a sesamoid bone in the FCU muscle, entails its unique vulnerability to distinct mechanisms of injury, as well as specific nonoperative clinical (NPC) challenges [14]. In this particular case, the transverse fracture pattern seen in the X-rays, in conjunction with the fall, likely stems from compressive and tensile forces consistent with a fall onto an outstretched hand. This correlates with the two primary mechanisms of injury defined in the literature: direct trauma to the hypothenar eminence with wrist hyperextension, and avulsion forces from the FCU tendon during falls [6,7]. Such an injury mechanism elucidates why pisiform fractures tend to undergo significant trauma while clinical details remain often understated, resulting in a need for elevated clinical suspicion from the physician attending patients with ulnar-sided wrist pain [8].

Because of the bone’s diminutive dimensions and the frequent overlapping by the triquetrum on basic radiographs, diagnostic imaging poses particular difficulties in pisiform fractures [9]. Although our patient’s fracture was identifiable on standard X-rays, there are claims that almost 30% of carpal fractures may be converted to advanced imaging due to the initial radiographic incongruities [15,16]. CT scans have become widely accepted for the identification of hidden fractures of the pisiform bone due to its near 100% sensitivity in some studies, compared to MRI, which assesses tears of soft tissue related to the joint better [3,11]. Our decision to restrict additional imaging was supported by the fracture being clearly visible on his plain films and clinical evidence of other injuries being absent. It was, however, a mistake not to approach this case with lower bounds for additional studies in non-congruous circumstances.

As shown by our patient’s outcome with a below-elbow ulnar gutter splint, conservative management with immobilization remains the backbone of treatment for non-displaced pisiform fractures [4]. In our case, the two-week period of progressive mobilization and immobilization was at odds with some literature recommendations of four to six weeks of casting, suggesting a lower bound on the compliance provided by the patient competent with non-displaced fractures. These fractures show a strong tendency towards compliance deformation [2,13,17]. This strategy mitigates the risks of stiffness, particularly critical in our military population patients, while enabling fracture stabilization. Our patients, who underwent functional assessments at four to six weeks postoperatively, demonstrated favorable outcomes, which bolstered the validity of this approach. Patients with modifiable risk factors for non-union, like smoking or diabetes, may still need more conservative immobilization protocols [10,18].

The need for proper management and effective treatment diagnostic methods is emphasized by the potential complications resulting from pisiform fractures. The chronic pisotriquetral pain that develops in approximately 15% of cases may lead to significant impairment in hand function, and if not adequately treated, conservative measures will fail, requiring subsequent pisiform excision [19]. Ulnar neuropathy is another worrisome complication, either resulting from the initial injury or inflicted as a surgical complication during later excision. There are some studies that indicate nearly 20% of excision cases experience transient neuropraxia [20]. The uncomplicated recovery in our patient illustrates the importance of early diagnosis and initial treatment relative to these sequelae, although practitioners must remain cautious for unmasked healing signs that suggest complication progression.

As isolated pisiform fractures are so rare, the evidence for their optimal management has been scant, and most available literature consists of case reports and small case series [5,6,8,9]. This case report contributes to this scarce literature by showing that short-term immobilization works in a young, healthy patient, while also emphasizing the need for the appropriate anatomical insight and clinical acumen necessary to make appropriate working diagnoses of these disorders. Subsequent work needs to focus on the creation of standardized diagnostic pathways and the prospective assessment of various immobilization strategies to assist clinical decision-making. In particular, these patients may be most affected by the occupational consequences of needing to resume manual work activities quickly, which raises the need to investigate the impacts of early mobilization versus an extended period of immobilization.

This case teaches us several lessons in the clinical management of pisiform fractures. The injury is often sustained from minimal trauma, and clinicians must remain highly suspicious even if the injury appears benign [5,10,21]. Advanced imaging can be indispensable in ambiguous cases, but careful interpretation of standard radiographs using multiple views can provide a diagnosis in more straightforward cases. Then, fractures should be treated according to their individual characteristics as well as patient-specific factors, which may allow some patients shorter periods of immobilization. Close follow-up is still necessary to monitor for complications after clinically healing, even when visible signs of recovery have taken place. Anatomical understanding alongside clinical detail capture aids in managing this rare injury.

## Conclusions

The case report highlights important perspectives regarding the diagnosis and management of isolated pisiform fractures, considering the rarity of such carpal injuries. Our case underscores the importance of clinical vigilance, complete imaging workup, and non-operative measures. Usually, pisiform fractures heal satisfactorily with immobilization of the wrist in a cast, but this should be tailored to the individual. Early diagnosis before symptoms develop, such as chronic pain or osteoarthritis in patients, stresses the timeliness of intervention. The report highlights the lack of prospective studies on the optimal guidelines for the prescription of dynamic and static immobilization, as well as the criteria for surgical procedure, warranting guidance. Thus, there should be increased attention by emergency physicians, orthopedic surgeons, and radiologists with respect to pisiform fractures while analyzing ulnar-sided wrist pain or any relevant ulnar wrist pathology to optimize the management of isolated pisiform fractures.
